# ﻿Two new pendulous epiphytic *Columnea* L. (Gesneriaceae) species from the Chocó forests of the Northern Andes

**DOI:** 10.3897/phytokeys.196.79673

**Published:** 2022-05-16

**Authors:** Francisco Tobar, James F. Smith, John L. Clark

**Affiliations:** 1 Área de Investigación y Monitoreo de Avifauna, Aves y Conservación – BirdLife en Ecuador, Quito, Ecuador Área de Investigación y Monitoreo de Avifauna, Aves y Conservación – BirdLife en Ecuador Quito Ecuador; 2 Instituto Nacional de Biodiversidad, Herbario Nacional del Ecuador QCNE, Quito, Ecuador Instituto Nacional de Biodiversidad, Herbario Nacional del Ecuador QCNE Quito Ecuador; 3 Department of Biological Sciences, Boise State University, 1910 University Drive, Boise, Idaho, 83725, USA Boise State University Boise United States of America; 4 Science Department, The Lawrenceville School, Lawrenceville, NJ 08648, USA Science Department, The Lawrenceville School Lawrenceville United States of America

**Keywords:** Chocó, Colombia, Columnea, Ecuador, Gesneriaceae, taxonomy

## Abstract

Exploratory field expeditions to the Chocó forests in the northwestern slopes of the Ecuadorian and Colombian Andes resulted in the discovery of two new species of *Columnea* (Gesneriaceae). *Columneafluidifolia* J.L.Clark & F.Tobar, **sp. nov.**, is described as a narrow endemic from Bosque Protector Mashpi and surrounding areas in the province of Pichincha in northern Ecuador. *Columneapendens* F.Tobar, J.L.Clark & J.F.Sm., **sp. nov.**, is described from recently discovered populations in the provinces of Carchi and Santo Domingo de los Tsáchilas (Ecuador) and the departments of Cauca and Nariño in southwestern Colombia. The two new species are pendent epiphytes with elongate shoots and shallowly bilabiate to nearly tubular corollas. Descriptions, complete specimen citations, and a distribution map are provided. Based on IUCN guidelines, a preliminary conservation status of Critically Endangered (CR) is provided for *C.fluidifolia* and Endangered (EN) is provided for *C.pendens*.

## Abstract (es)

Las expediciones de campo a los bosques del Chocó en las laderas noroccidentales de los Andes de Ecuador y Colombia dieron como resultado el descubrimiento de dos nuevas especies de *Columnea* (Gesneriaceae). *Columneafluidifolia* J.L.Clark & F.Tobar, **sp. nov.**, descrita como endémica de una reducida área del Bosque Protector Mashpi y zonas circundantes en la provincia de Pichincha. *Columneapendens* F.Tobar, J.L.Clark & J.F.Sm., **sp. nov.**, se describe a partir de poblaciones recientemente descubiertas en las provincias de Carchi, Santo Domingo de los Tsáchilas (Ecuador) y los departamentos de Cauca y Nariño en el suroeste de Colombia. Las dos nuevas especies son epífitas pendulares con brotes alargados y corolas ligeramente bilabiadas a casi tubulares. Descripciones, citaciones completas de los especímenes y mapa de distribución son incluidas. Basados en los criterios de la IUCN, se asigna un estado de conservación preliminar de En Peligro Critico (CR) para *C.fluidifolia* y a *C.pendens* se le asigna la categoría de En Peligro (EN).

## ﻿Introduction

The flowering plant family Gesneriaceae, with more than 3400 species and 150 + genera (Weber 2004; Weber et al. 2013), is in the order Lamiales. The family is divided into three subfamilies and seven tribes (Weber et al. 2013, 2020), which represent monophyletic lineages (Ogutcen et al. 2021). The majority of New World members are in the subfamily Gesnerioideae and are represented by 1200+ species and 77 genera (Clark et al. 2020). *Columnea* L. is classified in the tribe Gesnerieae and subtribe Columneinae (Weber et al. 2013, 2020). The genus *Columnea* is distinguished by fruits that are indehiscent berries in contrast to fleshy bivalved capsules in closely related genera.

**Figure 1.**
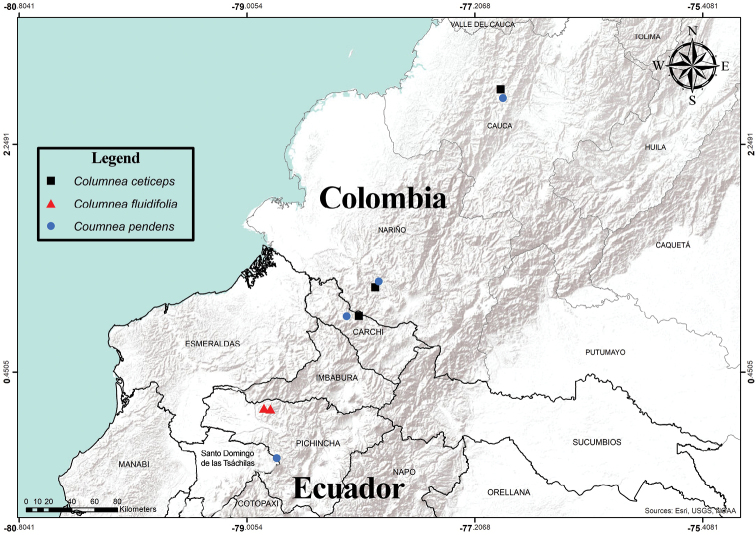
Distribution of *Columneaceticeps* (squares), *C.fluidifolia* (triangles), and *C.pendens* (circles). Note that range of *C.ceticeps* extends into the Colombian departments of Chocó, Antioquia and Risaralda (not featured here) (map provided by Marco F. Monteros).

Most *Columnea* are epiphytic with primary shoots that are characterized as erect, horizontal, dorsiventral (associated with facultative epiphytes), or pendent. The two species described here are characterized as epiphytes with elongate pendent shoots, a habit more typical of *Columnea* from Central America. In contrast, most *Columnea* in South America are facultative epiphytes with dorsiventral shoots. For example, Panama and Costa Rica have collectively more than 20 species of *Columnea* that are characterized as pendent epiphytes. Some examples of species in the northern Andes with elongate pendent shoots include *Columneabillbergiana* Beurl., *C.kienastiana* Regal., and *C.minor* Hanst. *Columneafluidifolia* is endemic to the Bosque Protector Mashpi and surrounding areas in the province of Pichincha in northern Ecuador (Fig. 1). *Columneapendens* is known from the provinces of Carchi and Santo Domingo de los Tsáchilas (Ecuador) and the departments of Cauca and Nariño in southwestern Colombia (Fig. 1). The two species described here have pendent shoots, a habit more typical for *Columnea* in Central America.

*Columnea* ranges from Mexico south to Bolivia, and is most diverse in the northern Andes of Colombia and Ecuador. With more than 210 species (Clark et al. 2020), *Columnea* is the largest genus in the subfamily Gesnerioideae (Weber et al. 2013, 2020). *Columnea* is strongly supported as a monophyletic genus based on molecular phylogenetic studies (Smith et al. 2013a; Schulte et al. 2014). Most subgeneric ranks are artificially defined and not supported by phylogenetic studies (Smith and Carroll 1997; Smith 2000; Clark and Zimmer 2003; Clark et al. 2012; Smith et al. 2013a; Schulte et al. 2014). Thus, we refrain from classifying the new species into a subgeneric rank.

## ﻿Materials and methods

Plants were photographed in the field and subsequently pressed and dried. Specimens are currently deposited at the National Herbarium of Ecuador (QCNE) and the herbarium at the Pontificia Universidad Católica del Ecuador (QCA). Additional specimens will be distributed to the Universidad Estatal Amazónica (ECUAMZ), Marie Selby Botanical Gardens (SEL) and the United States National Herbarium (US). Photographs were taken of live specimens in the field using a Nikon D100 DSLR with a Nikon 105 mm lens. Morphological observations and measurements were made from live collections, alcohol-preserved material, and digital images using the ImageJ program (https://imagej.nih.gov/ij/). Collections from the herbaria QCNE and QCA were consulted as well as type specimens from Jstor Global Plants (https://plants.jstor.org).

We assessed the extinction risk of the two new species following the IUCN Red List Categories and Criteria (2012). We considered observations, collection localities, and population estimates from fieldwork. Species extent of occurrence (EOO) and area of occupancy (AOO) were calculated using *GeoCAT* (Bachman et al. 2011; http://geocat.kew.org/) with the default setting of 2 km^2^ grid.

## ﻿Taxonomic treatment

### ﻿ Columnea fluidifolia

Taxon classificationPlantaeLamialesGesneriaceae

J.L.Clark & F.Tobar sp. nov.

078AF250-231E-51F8-AFAB-447BCD8A1B72

urn:lsid:ipni.org:names:77297807-1

Figs 2 , 3

#### Type.

Ecuador. Pichincha: cantón Quito, parroquia Pacto, primary road between the town of Pacto and Mashpi Lodge, 0°9'49.3"N, 78°49'14.6"W, 1662 m, 15 Mar 2019, *J.L. Clark & L. Jost 16286* (holotype: US; isotypes: ECUAMZ, QCA, SEL).

#### Diagnosis.

Similar to *Columneaceticeps*, differing in calyx and corolla uniformly orange (vs. calyx green and corolla bright red in *C.ceticeps*) and corolla shallowly bilabiate (vs. deeply bilabiate in *C.ceticeps*).

#### Description.

Epiphytic herb with elongate pendent shoots, 1.0–1.5 m long, red-brown, with zigzag appearance, densely pilose with gold-colored multicellular trichomes; internodes 2.0–4.0 cm long. Petioles 0.2–0.4 cm long, pilose with multicellular trichomes; leaves opposite, pairs either strongly anisophyllous or isophyllous, sometimes anisophyllous and isophyllous on the same shoot, larger leaf 9.5–10.5 cm long, 3.9–4.5 cm wide, ovate-elliptic, apex long acuminate, base slightly oblique, lateral veins 5–8 per side, adaxially dark-green, with multicellular white-transparent trichomes, abaxially light-green, densely pilose with multicellular white transparent trichomes, more densely pubescent on veins, margin serrulate; smaller leaf 1.3–1.9 cm long, 0.2–0.3 cm wide, lateral veins 2–3 per side, petiole 0.1–0.2 cm long, otherwise similar to larger leaf. Inflorescence reduced to a single axillary flower; bracts not seen, presumably caducous. Pedicels 2.6–3.7 cm long, bright orange, densely pilose with multicellular rust-colored trichomes. Calyx loosely clasping corolla, uniformly bright orange, lobes 2.6–4.2 cm long, 0.4–0.6 cm wide, oblong to narrowly-elliptic, apex acuminate to obtusely acuminate, exterior pilose, with multicellular rust-colored trichomes, more pubescent on veins and margins, interior glabrous, margin serrulate. Corolla 4.5–4.9 cm long, 0.6–0.8 cm at widest point, tubular throughout, inflated near center, slightly gibbous at the base, 0.6–0.7 cm wide before the limb, 0.2–0.4 cm at narrowest point of the base, bright orange externally, densely pubescent with multicellular rust-colored trichomes, interior with short white trichomes; ventral and lateral lobes reflexed, ventral lobe narrowly triangular to oblong, 0.4–0.5 cm long 0.2–0.3 cm wide, dorsal lobes fused, 0.2–0.3 cm long, 0.4–0.5 cm wide, lateral lobes triangular to narrowly triangular, 0.2–0.3 cm long, 0.2–0.3 cm wide. Filaments ca. 4.5 cm long, connate at base for 0.3 cm and adnate to corolla, anthers ca. 1.0 mm long, 1.0 mm wide, included in the corolla throat, quadrangular. Ovary ca. 4.0 mm long, conical, densely pubescent, with multicellular transparent trichomes; style 3.5–4.0 cm long, white with yellow apex, glabrescent; stigma unlobed, papillate, included in corolla tube. Nectary a bilobed dorsal gland. Fruit an indehiscent succulent berry, globose, distally acuminate, uniformly white, pilose; seeds not seen.

#### Phenology.

Flowering during June, August, and November. Mature fruits observed in June.

#### Etymology.

**Figure 2.**
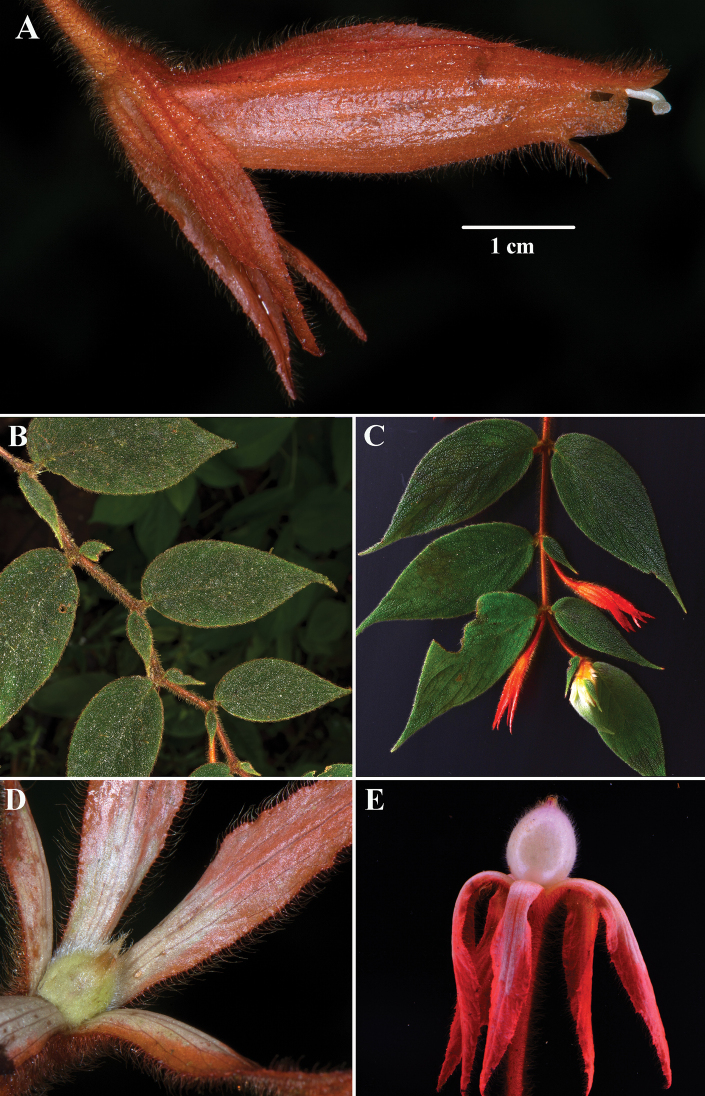
*Columneafluidifolia* J.L.Clark & F.Tobar **A** lateral view of mature flower **B** anisophyllous leaf pairs **C** isophyllous leaf pairs (base & apex) and anisophyllous leaf pairs (middle) **D** immature berry **E** mature berry (**A, B, D** from *J.L. Clark 16286***C, E** from *A.J. Perez & F. Tobar sn*). Photos **A, B, D** by J.L. Clark & **C, E** by F. Tobar.

The phyllotaxy in most neotropical Gesneriaceae is opposite with pairs that are either equal in size (isophyllous) or strongly unequal in size (anisophyllous). The leaves in *C.fluidifolia* are unusual for having anisophyllous leaf pairs (Fig. 2B) and isophyllous leaf pairs (Fig. 2C) on the same shoot. The specific epithet reflects the remarkable variability of this vegetative character in *C.fluidifolia*.

#### Distribution and preliminary assessment of conservation status.

*Columneafluidifolia* is endemic to the northwestern Andean slopes of Ecuador. The only known populations are located in the Mashpi-Pachijal conservation area, in the northwestern province of Pichincha (Fig. 1). The Mashpi-Pachijal conservation area is located in the broadly defined ecoregion referred to as the Chocó Biogeographic Region or the Tumbes-Chocó-Magdalena biodiversity hotspot. According to the vegetation classification system by the Ecosystems of Continental Ecuador (MAE 2012), the vegetation is classified as *Bosque siempreverde piemontano de Cordillera Occidental de los Andes* (*BsPn01*) (premontane evergreen forest) and represents a narrow band between 300 and 1400 meters, that ranges from the Esmeraldas province in the north to the Santo Domingo de los Tsáchilas province in the south. Most remnants of this vegetation have been converted to agriculture crops or livestock. Fewer than 40 individuals of *C.fluidfolia* are known, with a majority in the privately owned reserves of Mashpi and Amagusa. Additional populations of *C.fluidifolia* are known along the primary road that connects the two reserves. Based on the available information and according to the IUCN Red List criteria (IUCN 2012; IUCN Standards and Petitions Committee 2019) *C.fluidifolia* is preliminarily assessed as Critically Endangered (CR) based on an EOO < 100 km^2^ (B1), limited geographic range (criteria B1 + 2a, b), and restricted population of fewer than 50 individuals (D).

**Figure 3.**
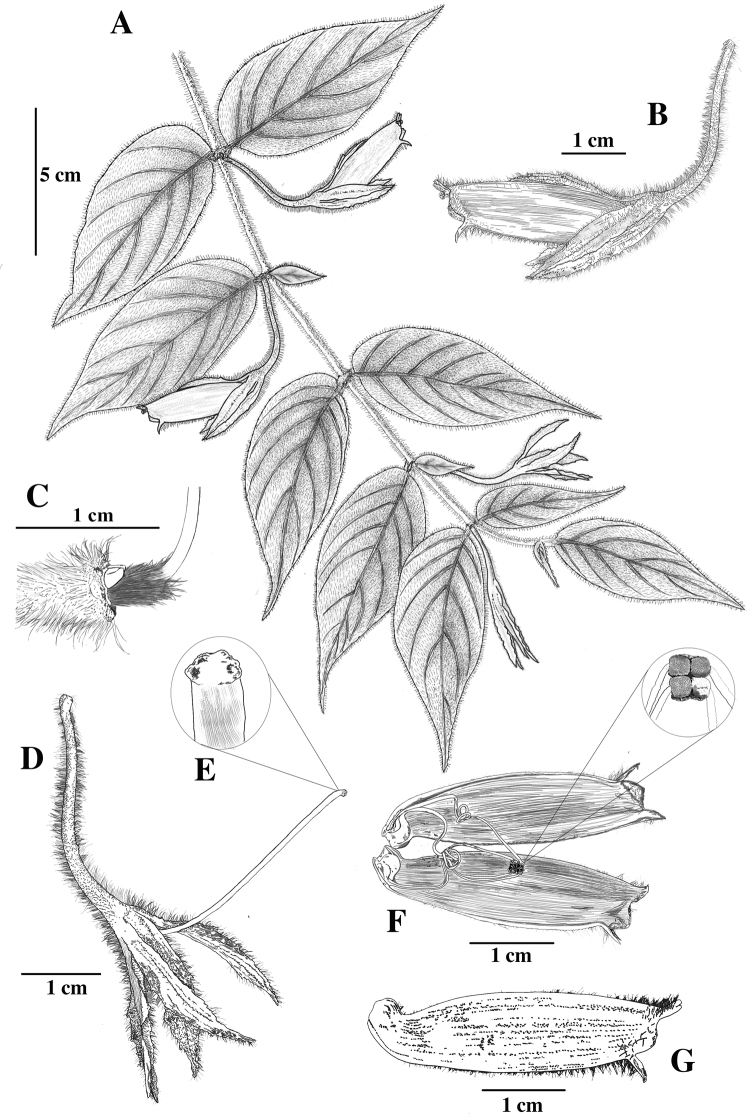
*Columneafluidifolia* J.L.Clark & F.Tobar **A** pendent shoot **B** lateral view of flower **C** bilobed dorsal nectary gland and ovary **D** mature calyx with style **E** stomatomorphic stigma **F** corolla opened featuring mature anthers **G** mature corolla. Illustration by M.J. Gavilanes.

#### Comments.

**Figure 4.**
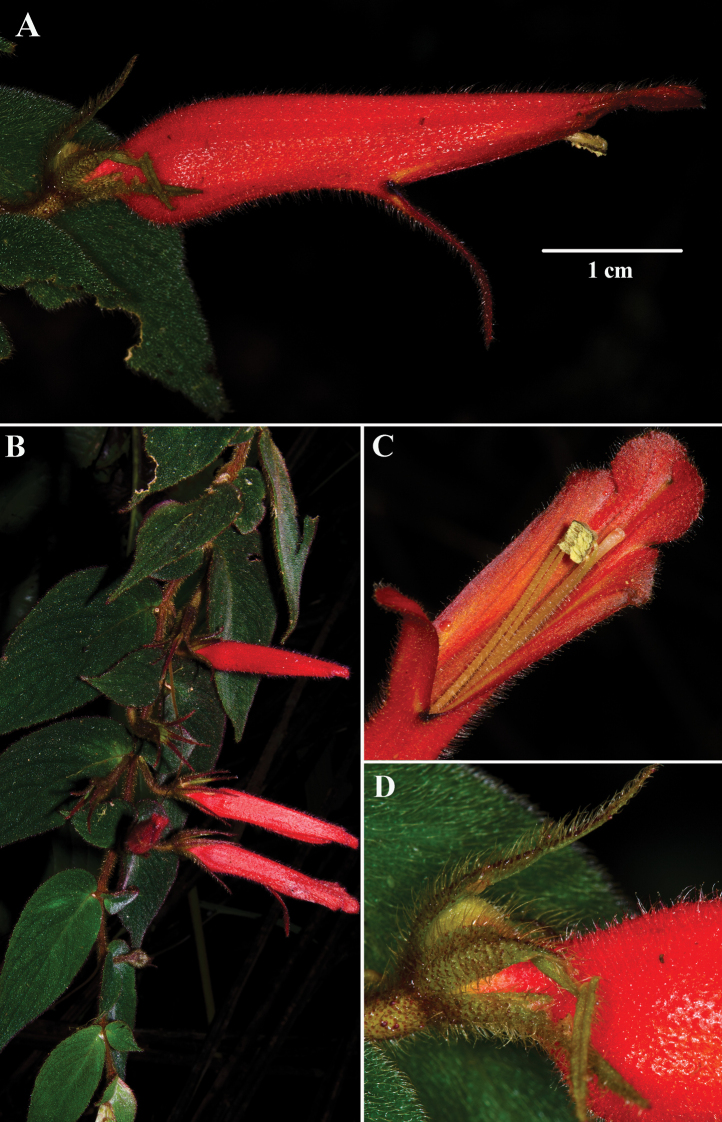
*Columneaceticeps* J.L.Clark & J.F.Sm. **A** mature flower **B** pendent shoot **C** deeply bilabiate corolla **D** calyx lobes (**A–D** from *J.L. Clark 12950***D** photos by J.L. Clark.

*Columneafluidifolia* differs from most *Columnea* by the presence of pendent elongate shoots and paired leaves that are both anisophyllous (Fig. 2A) and isophyllous (Fig. 2B). In addition, the shallowly bilabiate to tubular corolla and relatively large leaves (> 4 cm long) are not typical for other *Columnea* with pendent elongate shoots. In contrast, most *Columnea* with pendent elongate shoots have corollas that are deeply bilabiate and small leaves (< 4 cm long). *Columneafluidifolia* is similar to *C.ceticeps*. The calyx lobes and corolla are orange in *C.fluidifolia* (Fig. 2). In contrast, the calyx lobes are green, and the corolla is bright orange in *C.ceticeps* (Fig. 4). The corolla tube in *C.fluidifolia* is nearly tubular without the presence of a bilabiate limb (Figs 2, 3). In contrast, the corolla tube in *C.ceticeps* is deeply bilabiate (Fig. 4).

Ecuador. **Pichincha**: Distrito Metropolitano de Quito, Bosque protector Mashpi, Trasecto Mashpi Laguna 0.169750°N, 78.872120°W, 1150 m, 27 Jun 2017, *F. Tobar et al. 2908* (QCA); Bosque protector Mashpi, Trasecto Mashpi Laguna 0.169750°N, 78.872120°W, 1150 m, 21 Sep 2018, *F. Tobar et al. 3267* (QCA).

### ﻿ Columnea pendens

Taxon classificationPlantaeLamialesGesneriaceae

F.Tobar, J.L.Clark & J.F.Sm. sp. nov.

BAE52126-998A-5956-A510-26A05B9B7285

urn:lsid:ipni.org:names:77297808-1

Fig. 5

#### Type.

Ecuador. Carchi, Espejo, road between Goaltal and Chical, near to río Gualpi, 0.890555°N, 78.2188889°W, 1786 m, 12 Sep 2021, *F. Tobar 3638* (holotype: QCA; isotype: QCNE).

#### Diagnosis.

Similar to *Columneaceticeps*, differing in the bright orange corollas (vs. bright red corollas in *C.ceticeps*), calyx lobes broadly ovate (vs. narrowly elongate in *C.ceticeps*), corolla tube ventricose (vs. uniformly tubular in *C.ceticeps*), and corolla tube densely pubescent (vs. corolla tube sparsely pubescent in *C.ceticeps*).

#### Description.

Epiphytic herb with elongate pendent shoots, 1.2–1.5 m long, brown, with zigzag appearance, glabrous near base, pilose with gold-colored trichomes near apex; internodes 2.0–4.5 cm long. Petioles 0.1–0.2 cm long, pilose with multicellular gold-colored trichomes; leaves opposite, pairs usually, nearly equal to isophyllous, rarely anisophyllous, 0.6–6.0 cm long, 0.3–2.3 cm wide, ovate to elliptic, apex acuminate, base slightly oblique, lateral veins 3–8 per side, adaxially dark-green, with white trichomes, abaxially green to red-purple, densely pilose with multicellular gold-colored trichomes and single-celled white trichomes, margin crenulate to serrulate. Inflorescence reduced to 1 or 2 flowers per axil; bracts not seen, presumably caducous. Pedicels 0.3–1.3 cm long, green or red, appressed pilose with multicellular gold-colored trichomes. Calyx clasping, lobes 1.2–2.3 cm long, 0.25 cm wide, triangular-ovate to broadly triangular-ovate, apex long acuminate, exterior densely pilose, with multicellular gold-colored trichomes, interior glabrous, margin serrulate. Corolla 5.2–6.1 cm long, 0.6–1.1 cm at widest point (apex of corolla limb, near throat), tubular, medially ventricose, gibbous at base, 2.8–3.5 mm wide at narrowest point at the base, bright orange externally, interior yellow, exterior densely pubescent with multicellular red-colored trichomes, interior with sparse short trichomes and some stalked glandular trichomes; limb bilabiate, upper lip with two fused dorsal lobes and two lateral lobes, lower limb with an extended ventral lip; dorsal lobes connate, rounded to subquadrate, ca. 6.2 mm long, ca. 6.5 mm wide; lateral lobes triangular to narrowly triangular, ca. 1.5 mm long, ca. 2.0 mm wide; ventral lobe, narrowly oblong to linear ca. 1.9 cm long and ca. 2.8 mm wide, galea 1.9 cm long. Filaments ca. 5.1 cm long, connate for ca. 0.5 cm and adnate to base of corolla, anthers ca. 1.4 mm long, ca. 1.3 mm wide, included in the corolla throat, quadrangular. Ovary 3.4 mm long, conical, pubescent or glabrous, with multicellular transparent trichomes; style pale yellow to white, pilose with multicellular transparent and short stalked glandular trichomes; stigma unlobed, papillate, included in corolla tube. Nectary a bilobed dorsal gland. Mature fruit and seeds not seen.

#### Phenology.

Collected in flower during February-April, and September. Immature fruits observed in April.

#### Etymology.

The specific epithet refers to the pendent epiphytic habit.

#### Distribution and preliminary assessment of conservation status.

*Columneapendens* is endemic to the western Andean slopes of Ecuador and Colombia (Fig. 1). The forest is located in the broadly defined ecoregion referred to as the Chocó Biogeographic Region or the Tumbes-Chocó-Magdalena biodiversity hotspot.

The Ecuadorian populations are known from the provinces of Santo Domingo de los Tsáchilas and Carchi (Fig. 1). The Ecuadorian population from Santo Domingo de los Tsáchilas is located on the road that connects the village 23 de Julio with San Juan de Chiriboga. Several populations from Carchi are located near Río Gualpi, between 1500 and 1800 m. According to the vegetation classification system by the Ecosystems of Continental Ecuador (MAE 2012), the Ecuadorian forest is classified as *Bosque siempreverde montano bajo de Cordillera Occidental de los Andes* (*BsBn04*) (lower montane evergreen) and represents a narrow band of vegetation between 1400 and 2000 meters, that ranges from the Carchi province in the north to the Santo Domingo de los Tsáchilas province in the south. Current protected areas in Ecuador that correspond to montane evergreen forest include the Ecuadorian national park (Ministerio del Ambiente del Ecuador), Cotacahi-Cayapas Ecological Reserve, and several private reserves such as Los Cedros Biological Reserve, Maquipucuna Cloud Forest Reserve, and Río Guajalito.

The Colombia populations are known from Cerro Plateado, Cerro Pinche, and Munchique in the Cauca department, and La Planada in the Nariño department (Fig. 1). The Colombian forests that host populations of *C.pendens* correspond to premontane, montane, pluvial, and cloud forests along the Pacific slopes of the Cordillera Occidental (western mountain range). Current protected areas in Colombia that correspond to known populations of *C.pendens* include the community-based reserve, La Reserva Natural La Planada, in the Nariño department in southern Colombia. In addition, populations of *C.pendens* are located in the Munchique National Natural Park, part of the Colombian Sistema Nacional de Áreas Protegidas, in the Cauca department.

Based on the available information and According to the IUCN Red List criteria (IUCN 2012; IUCN Standards and Petitions Committee 2019) *C.pendens* is preliminary assessed as Endangered (EN) based on fewer than five known populations (criteria B1 + 2a), EOO < 5000 km^2^, and AOO < 500 km^2^.

#### Comments.

**Figure 5.**
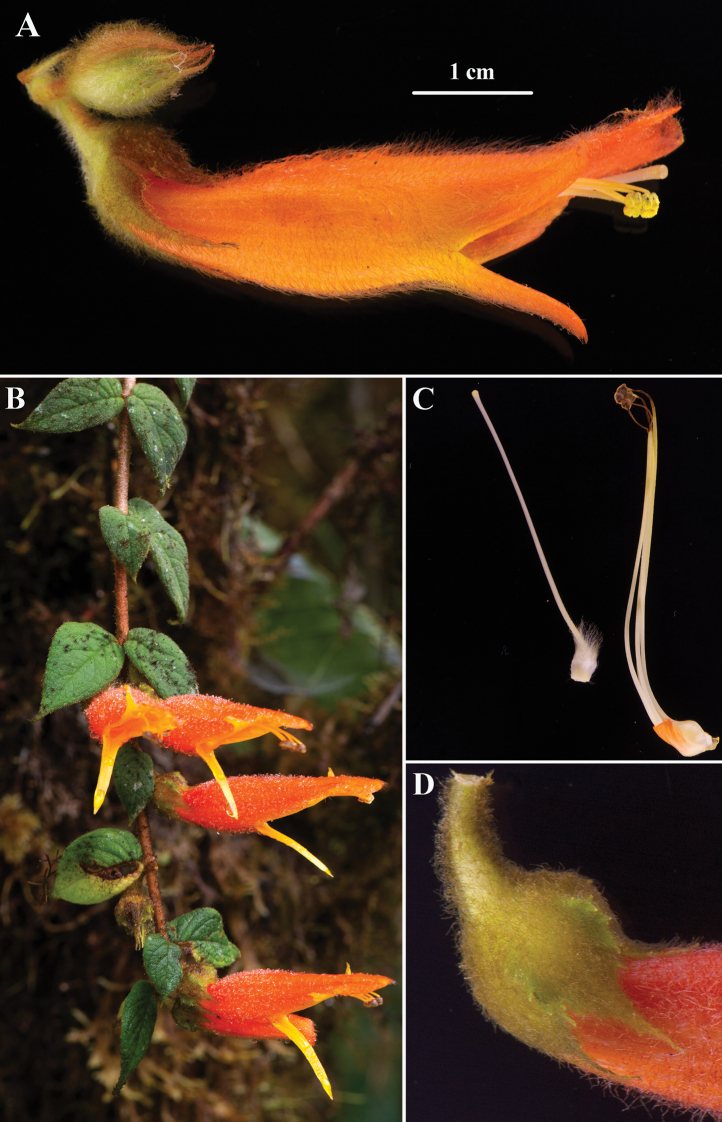
*Columneapendens* F.Tobar, J.L.Clark & J.F.Sm. **A** mature flower **B** pendent shoot **C** gynoecium and androecium **D** calyx lobes (**A–D** from *F. Tobar* 3638). Photos by F. Tobar.

*Columneapendens* is similar to *C.ceticeps*, which was recently described from Colombia and northern Ecuador (Smith et al. 2013b). The geographic distribution for these two species overlaps in northern Ecuador and southern Colombia (Fig. 1). *Columneapendens* has uniformly bright orange corollas (Fig. 5A) in contrast to the uniformly bright red corollas in *C.ceticeps* (Fig. 4A). The calyx lobes of *C.pendens* are broadly ovate (Fig. 5A) in contrast to the narrowly elongate calyx lobes of *C.ceticeps* (Fig. 4A). The corolla in *C.pendens* is medially inflated or ventricose and covered in uniformly dense (nearly tomentose) pubescence (Fig. 5A) in contrast to a sparsely pubescent corolla in *C.ceticeps* (Fig. 4A).

#### Specimens examined.

Colombia. **Cauca**: toward south end of La Depresion, between Cerro Pinche and Cerro Plateado, 26 Sep 1994, *E.L. Core 1358* (US); El Tambo, *O. Haught 5200* (US); El Tambo, Munchique Jul 1948 *S. Yepes Agredo 472* (US). **Nariño**: Reserva Natural La Planada, Ricaurte, on the road near Quebrada Dulce, 3 Mar 1989, *J.F. Smith & P. Galeano 1522*, (COL, WIS). **Ecuador. Carchi**: canton Mira, via Gualchan-El Carmen-Chical, 0.4166667°N, 78.216666°W, 1900–2000 m, 2 Nov 2014, *AJ Perez et al. 7716* (QCA). **Santo Domingo de los Tsáchilas**: cantón Santo Domingo, road between 23 de Julio y San Juan de Chirboga, 0.239444°S, 78.848611°W, 1891 m, 21 April 2021, *F. Tobar & G. Rueda 3644* (QCA).

## Supplementary Material

XML Treatment for Columnea fluidifolia

XML Treatment for Columnea pendens

## References

[B1] Bachman S Moat J Hill A de la Torre J Scott B (2011) Supporting Red List threat assessments with GeoCAT: Geospatial conservation assessment tool. ZooKeys 150: 117–126. 10.3897/zookeys.150.2109 PMC3234434 22207809

[B2] Clark JL Zimmer EA (2003) A preliminary phylogeny of *Alloplectus* (Gesneriaceae): Implications for the evolution of flower resupination. Systematic Botany 28: 365–375.

[B3] Clark JL Funke MM Duffy AM Smith JF (2012) Phylogeny of a Neotropical clade in the Gesneriaceae: More tales of convergent evolution. International Journal of Plant Sciences 173(8): 894–916. 10.1086/667229

[B4] Clark JL Skog LE Boggan JK Ginzbarg S (2020) Index to names of New World members of the Gesneriaceae (Subfamilies Sanangoideae and Gesnerioideae). Rheedea 30: 190–256. 10.22244/rheedea.2020.30.01.14

[B5] IUCN (2012) IUCN Red List Categories and Criteria. Version 3.1, 2^nd^ edn. IUCN, Gland, Switzerland and Cambridge, UK.

[B6] IUCN Standards and Petitions Committee (2019) Guidelines for using the IUCN Red List Categories and Criteria. Version 14. [Downloadable from:] http://www.iucnredlist.org/documents/RedListGuidelines.pdf

[B7] MAE (2012) Ministerio del Ambiente del Ecuador. Sistema de Clasificación de los Ecosistemas del Ecuador Continental. Quito, Ecuador.

[B8] Ogutcen E Christe D Nishii K Salamin N Möller M Perret M (2021) Phylogenomics of Gesneriaceae using targeted capture of nuclear genes. Molecular Phylogenetics and Evolution 157: e107068. 10.1016/j.ympev.2021.107068 33422648

[B9] Schulte LJ Clark JL Novak SJ Ooi MT Smith JF (2014) Paraphyly of Section Stygnanthe (*Columnea*, Gesneriaceae) and a revision of the species of section Angustiflorae, a new section inferred from ITS and chloroplast DNA Data. Systematic Botany 39(2): 613–636. 10.1600/036364414X680861

[B10] Smith JF (2000) Phylogenetic resolution with the tribe Episcieae (Gesneriaceae): Congruence of ITS and *ndh*F sequences from parsimony and maximum-likelihood analyses. American Journal of Botany 87(6): 883–897. 10.2307/2656896 10860919

[B11] Smith JF Carroll CL (1997) A cladistic analysis of the tribe Episcieae (Gesneriaceae) based on *ndh*F sequences: Origin of morphological characters. Systematic Botany 22(4): 713–724. 10.2307/2419437

[B12] Smith JF Ooi MT Schulte LJ Amaya-Márquez M Pritchard R Clark JL (2013a) Searching for monophyly in the subgeneric classification systems of *Columnea* (Gesneriaceae). Selbyana 31: 126–142.

[B13] Smith JF Amaya-Márquez M Marín-Gómez OH Clark JL (2013b) Four new species of *Columnea* (Gesneriaceae) with primary distributions in Colombia. Journal of the Botanical Research Institute of Texas 7: 667–679.

[B14] Weber A (2004) Gesneriaceae. In: Kadereit J (Ed.) The Families and Genera of Vascular Plants.Vol. 7. Flowering Plants. Dicotyledons. Lamiales (Except Acanthaceae Including Avicenniaceae). Springer, Berlin, 63–158. 10.1007/978-3-642-18617-2_8

[B15] Weber A Clark JL Möller M (2013) A New Formal Classification of Gesneriaceae. Selbyana 31(2): 68–94.

[B16] Weber A Middleton DJ Clark JL Möller M (2020) Keys to the infrafamilial taxa and genera of Gesneriaceae. Rheedea 30: 5–47. 10.22244/rheedea.2020.30.01.02

